# A rare case of dysferlinopathy with paternal isodisomy for chromosome 2 determined by exome sequencing

**DOI:** 10.1002/mgg3.2110

**Published:** 2022-12-04

**Authors:** Huan Li, Liang Wang, Cheng Zhang

**Affiliations:** ^1^ Department of Neurology, National Key Clinical Department and Key Discipline of Neurology The First Affiliated Hospital, Sun Yat‐sen University Guangzhou China

**Keywords:** dysferlinopathy, exome sequencing, muscular dystrophy, paternal isodisomy

## Abstract

**Background:**

Dysferlinopathies are autosomal recessive muscular dystrophies resulting from defects in *DYSF* (MIM: 603009)*,* which is located on chromosome 2p13 and encodes the dysferlin protein.

**Methods:**

We performed exome sequencing and subsequent trio‐based analysis in a family with dysferlinopathy.

**Results:**

We report a young patient presenting with hyperCKemia and mild muscle weakness of the lower limbs. Exome sequencing of the proband revealed a homozygous frameshift mutation, NM_001130987.2:c.1471dupA(p.M491Nfs*15), in *DYSF*. The father was heterozygous for the mutation and the mother did not carry the mutation, as determined by genetic analyses, exome sequencing of parental samples, and a trio‐based analysis. Further analysis revealed that the *DYSF* gene was not deleted; instead, the entire chromosome 2 of the proband was inherited from the father. Thus, the child had paternal uniparental isodisomy for chromosome 2 (uniparental disomy [UPD]2 pat).

**Conclusion:**

We report the first case of dysferlinopathy caused by paternal isodisomy for chromosome 2. Furthermore, our findings highlight the importance of exome sequencing of the proband and parents and trio analyses in clinical settings, particularly when Mendelian inheritance cannot be confirmed, to identify the presence of UPD and to rule out large pathogenic deletions.

## Graphical abstract

We report a young patient presenting with hyperCKemia and mild muscle weakness of the lower limbs. Exome sequencing of the proband revealed a homozygous frameshift mutation, c.1471dupA(p.M491Nfs*15), in DYSF. The patient had paternal uniparental isodisomy for chromosome 2 (UPD2 pat). We report the first case of dysferlinopathy caused by paternal isodisomy for chromosome 2.

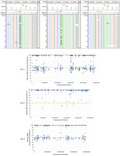

## 1 INTRODUCTION

Uniparental disomy (UPD) occurs when both homologous chromosomes are inherited from a single parent, that is, paternal UPD or maternal UPD. It can involve whole chromosomes or specific segments. According to the underlying mechanism, it can be divided into two types: heterodisomy (i.e., two different chromosomes come from a single parent) and isodisomy (i.e., a homologous chromosome pair includes two copies of the same chromosome from a single parent). Paternal UPD is rarer than maternal UPD (approximately 1:3), and heterodisomy is more frequent in maternal UPD, while isodisomy tends to occur in paternal UPD (Benn, 2021; Eggermann, 2020). Paternal UPD is rare for chromosome 2, with only nine reports, and all cases are uniparental isodisomy.

Dysferlinopathies are a group of autosomal recessive (AR) muscular dystrophies caused by mutations in the dysferlin gene (*DYSF*, MIM: 603009). *DYSF* is a large 150‐kb gene mapped to chromosome region 2p13. It encodes a 237‐kDa single‐pass transmembrane protein named dysferlin, which is located on the plasma membrane of skeletal muscle (Cárdenas et al., 2016). Dysferlin has an important role in the repair of muscle membrane lesions. Dysferlinopathies are characterized by varied and complicated phenotypes, which makes the confirmation of the absence of dysferlin in the muscle and the detection of *DYSF* mutations critical for their diagnosis. The two most common phenotypes are limb‐girdle muscular dystrophy type 2B (LGMD2B, MIM: 253601), starting with proximal muscle weakness of the lower limbs, and Miyoshi myopathy (MM, MIM:606768), which mainly affects the posterior compartment muscles of the leg (Illarioshkin et al., 2000). Other phenotypes have been described, such as distal anterior compartment myopathy, pseudometabolic form, proximodistal phenotype, asymptomatic hyperCKemia, and symptomatic carriers. The age at onset varies widely from congenital to 73 years (Klinge et al., 2008; Paradas et al., 2009), but it most commonly begins in the teenage or early adulthood, with complaints of lower limb weakness, difficulty climbing stairs and standing up from squatting, and, in some cases, muscle pain (Fanin & Angelini, 2016). Elevated serum creatine kinase (CK) levels (10–100 times normal values) are observed in all cases of dysferlinopathies and are usually observed from the asymptomatic stage. In addition, characteristic features of muscle magnetic resonance imaging (MRI) have been described in dysferlinopathies and can offer important clues for diagnosis; no difference of MRI patterns has been found with respect to phenotype (Jin et al., 2016; Paradas et al., 2010). In the asymptomatic stage, the finding of muscle edema may be the earliest signal of disease activity (Paradas et al., 2010).

In this report, we identified a 17‐year‐old male diagnosed with dysferlinopathy who had a homozygous mutation in the *DYSF* gene. In a follow‐up molecular analysis of parental DNA, we found that the homozygous mutation was inherited from the father due to paternal uniparental isodisomy of chromosome 2. To the best of our knowledge, this is the first case of dysferlinopathy resulting from paternal uniparental isodisomy of chromosome 2.

## 2 PATIENTS AND METHODS

### 2.1 Case presentation

**FIGURE 1:**
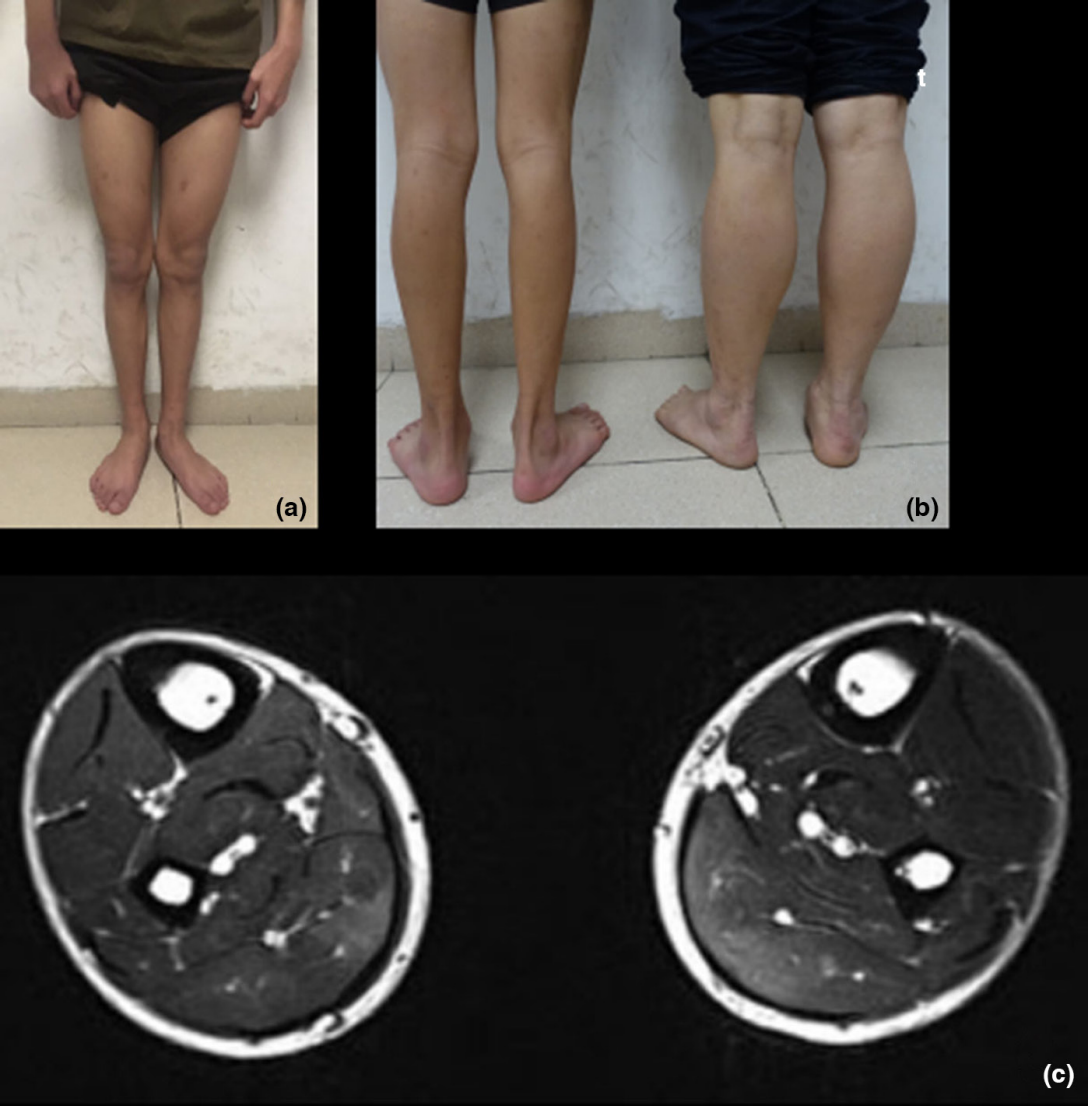
(a) Physical examination showed no atrophy of muscle, but the lower limbs of the patient (left) were thin, (b) comparison of the lower limbs of the father (right), (c) lower‐limb muscle magnetic resonance imaging showed increased signal for the bilateral soleus and musculi flexor pollicis longus on T2‐weighted images.

The patient was a 17‐year‐old male who came to our department after the finding of an elevated serum CK level of 8599 U/L (normal, <175 U/L) during preparation for surgery for left esotropia 1 month earlier. He was born to non‐consanguineous parents with a full‐term normal delivery. The birth weight was 3.2 kg. According to the parents, there were no delays in early motor milestones and no manifestation of mental retardation at an early age. The patient could walk independently and speak at age 1 year. No aberration of motor function was observed during growth. He had good performance in PE class and has the same ability to run and climb stairs as that of other individuals of the same age. A physical examination at 1 year of age showed that he had mild esotropia of the left eye and diplopia was not found. He was thin and long, thin feet were observed (Figure 1a,b). A manual muscle test indicated that the patient had normal muscle strength, and no obvious atrophy of the muscle was observed. He could walk on his toes, but performed worse when he was asked to walk on his heels. An electromyographic evaluation showed a pattern consistent with muscle lesions. An electrocardiogram and ultrasonic cardiogram indicated no cardiac relevance. MRI of the bilateral thighs did not show abnormalities, while MRI of the bilateral leg muscles showed an abnormal signal of the bilateral soleus and musculi flexor pollicis longus (increased signal on the T2‐weighted image and isointensity on the T1‐weighted image) (Figure 1c).

### 2.2 Molecular genetic study

Genomic DNAs of the proband and his parents were isolated from peripheral blood lymphocytes using the SolPure Blood DNA Kit (Magen, Beijing, China). To identify the causal mutation, targeted next‐generation sequencing covering 552 reported hyperCKemia‐related genes was performed. Multiplex primer pools were designed using Ion AmpliSeq™ Designer software (Life Technologies, Carlsbad, CA, USA). Genomic DNA reference sequences of the *DYSF* (NM_001130987.2) gene were obtained from the University of California, Santa Cruz (UCSC) Genome Browser database (http://genome.ucsc.edu/). Enrichment of exonic sequences was performed using the HTP Library Preparation Kit (KAPA Biosystems, Wilmington, MA, USA) and sequencing was performed using the Illumina NextSeq 500 system according to the manufacturer's protocol. Subsequent Sanger sequencing of genomic DNA was performed on samples obtained from the proband and his parents with the forward primer (5′‐CAGTGGAACCAGAACATCACA‐3′) and reverse primer (5′‐ATCCCTGGCTACCCCAAG‐3′) to confirm the detected variants. To investigate why the variant cannot be explained by Mendelian inheritance, whole‐exome sequencing was performed using the genomic DNA of the proband and his parents to identify single nucleotide variants or small insertion/deletions (InDels). Genomic DNAs were enriched using the SureSelect Human All Exon 50 Mb Kit (Agilent, Santa Clara, CA, USA). The resulting 350–400‐bp amplicons were then ligated to Proton adapters and purified according to the manufacturer's instructions (Ion Torrent, Gilford, NH, USA). Libraries were quantified by quantitative PCR and then loaded onto the NextSeq 500 sequencer for high‐throughput sequencing.

## 3 RESULTS

The homozygous mutation NM_001130987.2:c.1471dupA(p.M491Nfs*15) in *DYSF*, a previously described pathogenic mutation (Xi et al., 2014), was identified in the patient by targeted next‐generation sequencing. Further Sanger sequencing of both the proband and his parents confirmed the mutation in the proband and showed that the father was heterozygous for NM_001130987.2:c.1471dupA(p.M491Nfs*15) in *DYSF*, while the maternal genome lacked the mutation. These results suggested either the presence of a large deletion encompassing the *DYSF* locus on the maternal allele, UPD, or non‐maternity. The potential for non‐maternity was absolutely denied by the parents.

**FIGURE 2:**
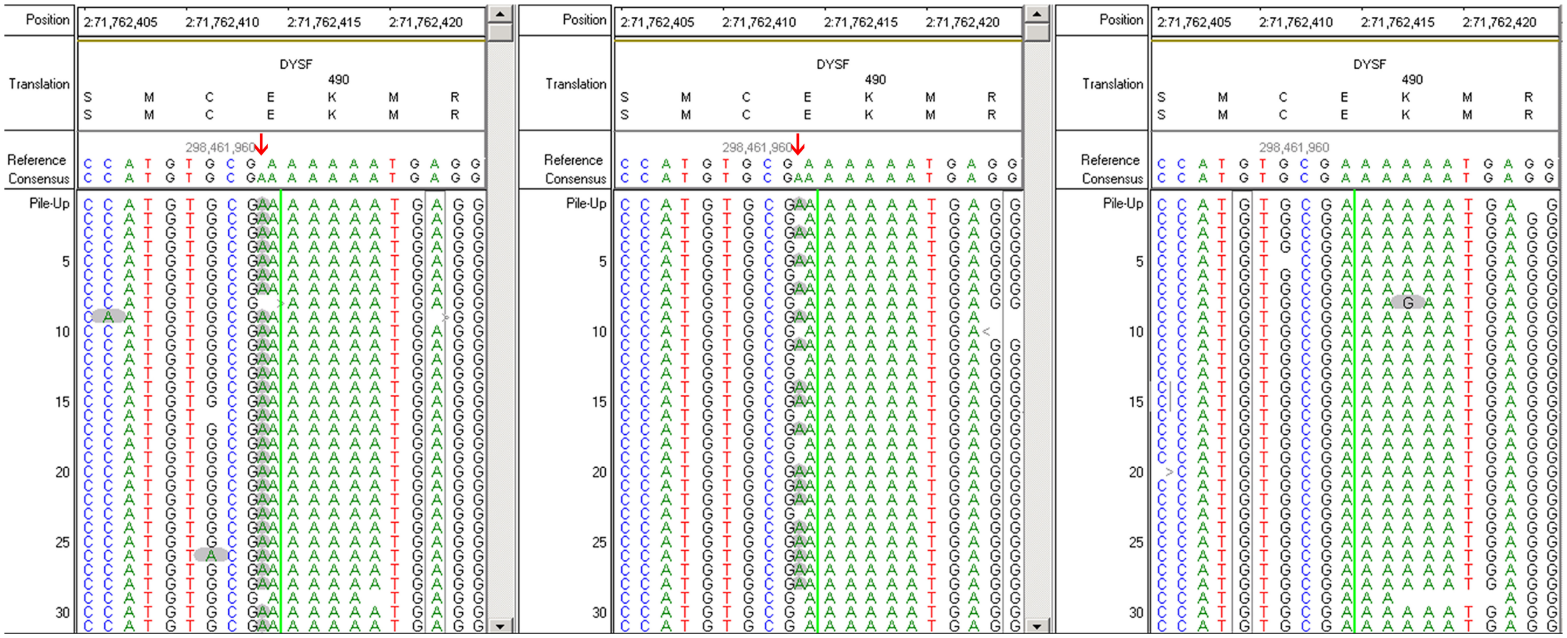
Whole‐exome sequencing confirmed the homozygous mutation of NM_001130987.2:c.1471dupA(p.M491Nfs*15) located at human GRCh37/hg19 chr2:71,762,414 (indicated by red arrows) in the patient (left), a heterozygous mutation in the father (middle), and no such mutation in the maternal genome (right).

**FIGURE 3:**
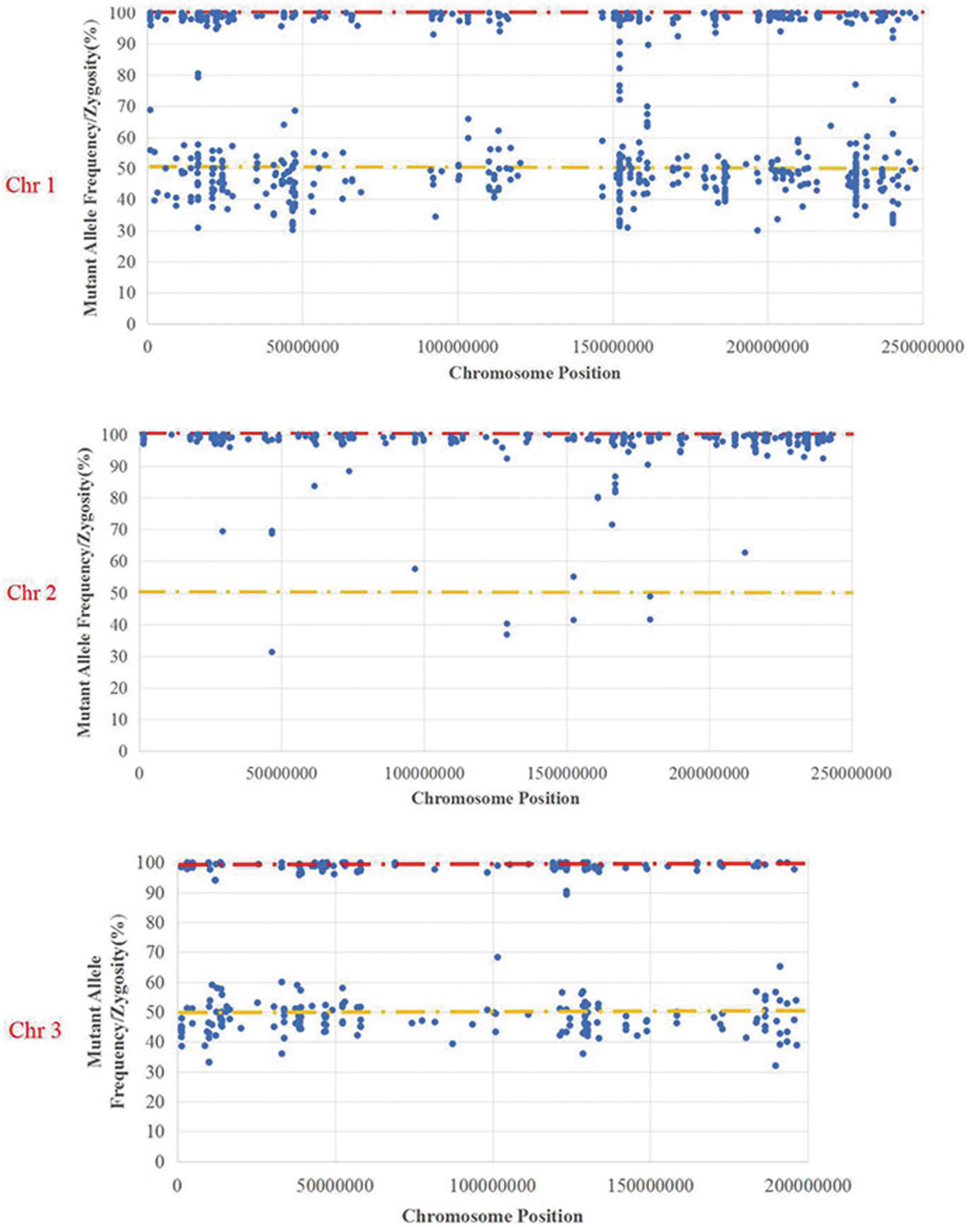
The mutant allele frequencies of chromosomes 1, 2, and 3 in the proband showed that chromosome 2 was almost completely homozygous along the whole length, and a similar pattern was not observed along chromosomes 1 and 3.

To clarify the genetic basis, we conducted next‐generation sequencing by whole‐exome sequencing in the parents and proband. These results confirmed the heterozygous c.1471dupA mutation of *DYSF* in the father and the lack of significant variants in the mother (Figure 2). Finally, we also analyzed the detected nucleotide polymorphisms in the patient and his parents in chromosome 2 by mapping to the published human reference genome, Human GRCh37/hg19, after filtering reads with low sequencing quality (read balance <0.15). The entire chromosome 2 of the patient was homozygous, and this pattern was not observed for other chromosomes, including chromosomes 1 and 3 (Figure 3). Among the 326 homozygous mutations identified in chromosome 2 of the proband, 140 mutations could not be explained by Mendelian inheritance and were entirely of paternal origin; these were distributed along chromosome 2, with locations from bp 148,115 to 242,066,830. There were no Mendelian errors among homozygous mutations in chromosomes 1 and 3 of the proband, which also excludes nonmaternity.

## 4 DISCUSSION

The diagnosis of dysferlinopathy was based on clinical manifestations, biochemical analyses (serum CK, 8599 U/L), characteristic aberrations of the bilateral leg muscles by MRI, and genetic analyses. MRI findings of the bilateral leg muscles indicated edema of characteristic muscles, illustrating disease activity. Since the patient did not show obvious symptoms of muscle weakness and wasting, the exact phenotype is unclear. However, distal involvement is unlikely, suggesting that MM can be ruled out. Further observation of the disease progression is needed to confirm the dysferlinopathy phenotype.

We report a case of dysferlinopathy caused by homozygous NM_001130987.2:c.1471dupA(p.M491Nfs*15) in *DYSF*, a frameshift mutation reported to result in LGMD2B (Xi et al., 2014). Whole‐exome sequencing of the proband showed the complete homozygosity of chromosome 2, indicating isodisomy. Mendelian errors of completely paternal origin confirmed paternal UPD and nonmaternity was excluded by analyses of autosomes other than chromosome 2. UPD is caused by aberrations during meiosis and/or mitosis, for example, trisomic reduction to disomy, monosomy rescue, gamete complementation, and post‐fertilization error. In our patient, trisomy reduction and gamete complementation are unlikely; both mechanisms are expected to result in some heterozygosity (Robinson, 2000). We believe that the observed paternal isodisomy is most likely caused by monosomy rescue.

The mechanisms of UPD that lead to clinical phenotypes are related to the homozygosity of autosomal recessively inherited mutations, prenatal or postnatal trisomy mosaicism, and genomic imprinting (Benn, 2021; Eggermann, 2020). UPD for chromosome 2 is rare. Among several previously reported cases of paternal UPD for chromosome 2, most of them exhibited AR disorders (Kohl et al., 2021; Sezer et al., 2020). The cases reported by Keller et al. (2009), Ou et al. (2013), and Chen et al. (2019) were phenotypically normal (Chen et al., 2019; Keller et al., 2009; Ou et al., 2013), and apart from the phenotypes of AR disorders, the six other cases were essentially phenotypically normal, suggesting that genomic imprinting leading to abnormalities of growth and development has little or no effect on the clinical consequences of paternal UPD for chromosome 2. Our patient suffered from left esotropia since he was 1 year old, apart from the AR disorder; this phenotype has not been reported in previous cases of paternal UPD of chromosome 2. It is difficult to estimate whether infantile esotropia in our patient is associated with UPD of chromosome 2 or is simply the result of a muscular disorder, since there are no other supporting cases. However, genomic imprinting is likely to cause a more severe outcome, with abnormal growth and development, and esotropia has been associated with some muscular disorders, like myotonic dystrophy (Bollinger et al., 2008), though there are no reports of this phenotype in dysferlinopathy, suggesting that esotropia is not associated with genomic imprinting on chromosome 2.

In conclusion, we present the first known case of dysferlinopathy associated with paternal UPD 2. We believe that whole‐exome sequencing of patients and parents is beneficial for patients in whom Mendelian inheritance cannot be confirmed, helping to identify the presence of UPD and rule out larger pathogenic deletions.

## AUTHOR CONTRIBUTIONS

Huan Li: collection and/or assembly of the case, manuscript writing; Liang Wang: collection and/or assembly of data, data analysis; Cheng Zhang: financial support, final approval of manuscript.

## FUNDING INFORMATION

This study was supported by grants from the Natural Science Foundation of China (grant nos. 81771359, 81471280, and 81271401), the National Natural Science Foundation of Youth Science Foundation (grant no. 81601087), the Guangzhou Science and Technology Plan (grant nos. 1561000153/201508020012) and Guangdong Provincial Key Laboratory for Diagnosis and Treatment of Major Neurological Diseases (No. 2014B030301035), the Southern China International Cooperation Base for Early Intervention and Functional Rehabilitation of Neurological Diseases (No. 2015B050501003), Guangzhou Clinical Research and Translational Center for Major Neurological Disease (No. 201604020010), and Guangdong Provincial Engineering Center for Major Neurological Disease Treatment.

## CONFLICT OF INTEREST

There are no conflicts of interest to declare.

## ETHICAL COMPLIANCE

The study was approved by Sun Yat‐Sen University and an informed consent form was acquired from the patient and his parents.

## Data Availability

The datasets supporting our findings are presented in the article.
